# Chestnut Shell Polyphenols Inhibit the Growth of Three Food-Spoilage Bacteria by Regulating Key Enzymes of Metabolism

**DOI:** 10.3390/foods12173312

**Published:** 2023-09-02

**Authors:** Xinfang Wang, Yue Li, Suwen Liu, Hao Wang, Xuedong Chang, Jingzheng Zhang

**Affiliations:** 1 College of Food Science & Technology, Hebei Normal University of Science and Technology, Qinhuangdao 066004, China; 2 Engineering Research Center of Chestnut Industry Technology of Ministry of Education, Hebei Normal University of Science and Technology, Qinhuangdao 066004, China; 3 State Key Laboratory of Food Nutrition and Safety, Tianjin University of Science and Technology (TUST), Tianjin 300457, China

**Keywords:** chestnut shell polyphenols, bacteriostasis, cell micromorphology, tricarboxylic acid cycle, molecular docking

## Abstract

The microbial contamination of food poses a threat to human health. Chestnut shells, which are byproducts of chestnut processing, contain polyphenols that exert various physiological effects, and thus have the potential to be used in food preservation. This study investigates the bacteriostatic effect and mechanism(s) of the action of chestnut shell polyphenols (CSPs) on three food-spoilage bacteria, namely *Bacillus subtilis*, *Pseudomonas fragi*, and *Escherichia coli*. To this end, the effect of CSPs on the ultrastructure of each bacterium was determined using scanning electron microscopy and transmission electron microscopy. Moreover, gene expression was analyzed using RT-qPCR. Subsequent molecular docking analysis was employed to elucidate the mechanism of action employed by CSPs via the inhibition of key enzymes. Ultrastructure analysis showed that CSPs damaged the bacterial cell wall and increased permeability. At 0.313 mg/mL, CSPs significantly increased the activity of alkaline phosphatase and lactate dehydrogenase, as well as protein leakage (*p* < 0.05), whereas the activity of the tricarboxylic acid (TCA) cycle enzymes, isocitrate dehydrogenase and α-ketoglutarate dehydrogenase, were inhibited (*p* < 0.05). The expression levels of the TCA-related genes *gltA*, *icd*, *sucA*, *atpA*, *citA*, *odhA*, *IS178_RS16090*, and *IS178_RS16290* are also significantly downregulated by CSP treatment (*p* < 0.05). Moreover, CSPs inhibit respiration and energy metabolism, including ATPase activity and adenosine triphosphate (ATP) synthesis (*p* < 0.05). Molecular docking determined that proanthocyanidins B1 and C1, the main components of CSPs, are responsible for the antibacterial activity. Therefore, as natural antibacterial substances, CSPs have considerable potential for development and application as natural food preservatives.

## 1. Introduction

Microbial activity is a key factor in food spoilage, which not only destroys the original nutritional value of food, but also produces toxins that pose a threat to human health. Currently, food-processing plants commonly use chemical synthetic preservatives and antibiotics, with low prices and remarkable anti-corrosion effects, to prevent food spoilage [1,2]; however, these pose potential safety concerns. The abuse of synthetic chemical preservatives may lead to health risks, such as respiratory diseases and infant and child growth retardation [3], while the excessive use of antibiotics may lead to drug resistance and excessive antibiotic residue [4,5]. Therefore, the search for, and development of, natural bacteriostatic agents to replace synthetic preservatives and antibiotics has become a research hotspot.

Natural bacteriostatic agents originate from a wide range of sources, including animals, microorganisms, plant extracts, and their derivatives. Protamine, an antimicrobial peptide extracted from the sperm cells of vertebrates, such as salmon, exhibits antibacterial effects, but its price is several times that of synthetic chemical preservatives, limiting its application [6]. Among natural microbial bacteriostatic agents, nisin is widely used in the food industry. However, owing to the particularities of its microbial structure and the complexity of the food environment, the bacteriostatic efficiency of nisin is relatively low, requiring combination with other preservatives [7]. Bacteriostatic agents derived from plant extracts are abundantly available [8]. Among these, phenolic compounds have attracted considerable attention due to their significant antibacterial activity [9]. Plant polyphenols can disrupt cell wall membrane permeability [10], and inhibit respiratory energy metabolism, protein function, genetic material synthesis, and normal bacterial growth and metabolism [11]. Yi et al. [12] found that tea polyphenols act primarily on the structure of the bacterial cell wall of *Pseudomonas aeruginosa*, increasing the permeability of the cell membrane, and causing the release of small proteins. The antibacterial activity of polyphenols depends largely on the structure of their phenolic hydroxyl groups, with different natural plant polyphenols exhibiting unique antibacterial activities. Therefore, a broad market exists for the identification and development of cheap natural plant polyphenol resources as natural preservative replacements.

**Figure 1:**
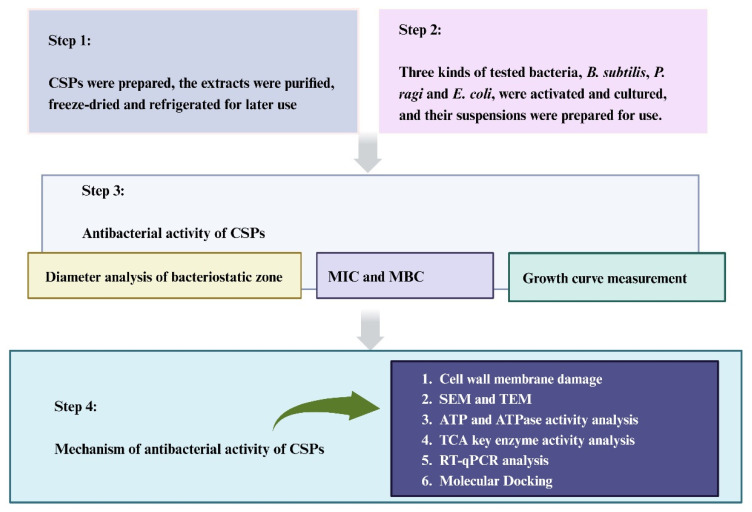
Research flow diagram for this study.

*Castanea mollissima* Blume (chestnut) belongs to the family Fagaceae and is an important edible nut in China, distributed primarily in the Yanshan and Taihang Mountains. Its cultivation history dates back 3000 years [13]. Chestnut shells are byproducts of chestnut processing that often accumulate and are burned as waste, resulting in resource wastage and environmental pollution [14]. However, while chestnut shells are rich in phenolic compounds and have anti-inflammatory, hypoglycemic, antioxidant, anticancer, and health-promoting effects [15], there are no reports on the antibacterial activity and mechanism of action of chestnut shell polyphenols (CSPs) against food-spoilage bacteria. Our previous study found that the main chemical components of CSPs are proanthocyanidin B1 (PB1) and proanthocyanidin C1 (PC1), which have significant effects on promoting the polarization of macrophages from the M1 to the M2 type, reducing inflammation and lowering lipid levels [16]. Therefore, it is speculated that CSPs may have a strong bacteriostatic effect; however, this effect and the associated mechanism remain unclear. Accordingly, the current study investigates the bacteriostatic mechanism of CSPs in terms of cell structure, enzyme activity, energy metabolism, and molecular gene expression. The research scheme is shown in Figure 1. The findings provide experimental data and theoretical support for the application of CSPs as natural preservatives in the food industry to effectively utilize Chinese chestnut resources.

## 2. Materials and Methods

### 2.1. Raw Material Preparation

Yanzi chestnuts were purchased from Qinglong County, Qinhuangdao City, Hebei Province, China (118°95′ E, 40°40′ N). The chestnut shells were dried in a 40 °C constant temperature blast-drying oven (DHG-9073A, Shanghai Shanzhi Instrument Preparation Co., Ltd., Shanghai, China), crushed, and sifted into 80 mesh powder (YB-500A, Yongkang Sufeng Industry & Trade Co., Ltd., Zhejiang, China). The powder was added to 70% anhydrous ethanol (1:7 ratio of material to liquid), extracted using ultrasonic treatment (KQ-500DE, Kunshan Ultrasonic Instrument Co., Ltd., Jiangsu, China) at 40 °C for 2 h, and then concentrated using vacuum filtration (EYELA, Elon Technology International Trade [Shanghai] Co., Ltd., Shanghai, China) at 40 °C. The concentrate was then purified using a macroporous resin (AB-8, Sigma-Aldrich, City of Saint Louis, MO, USA) and vacuum freeze-dried into a powder (LGJ-30D, Beijing Sihuan Scientific Instrument Factory Co., Ltd., Beijing, China) at −40 °C, sealed in brown glass tubes, and stored at −20 °C. As previously described [16], the polyphenol content of the extracted chestnut shells was 364.81 mg/g and contained 17 monomers, among which, PC1 and PB1 accounted for 34.5% and 40.6% of the total phenol content, respectively (Supplement Table S1).

*Bacillus subtilis*, *Pseudomonas fragi* (BNCC109047, BNCC134017; Henan Industrial Microbial Strains Engineering Research Center, Henan, China), and *Escherichia coli* ATCC25922 (Wuhan Miaoling Biotechnology Co., Ltd., Hubei, China) were used to evaluate CSP bacteriostasis. *B. subtilis* and *P. fragi* were uniformly coated in nutrient AGAR medium (NA) and cultured for 24 h at 30 °C in an oscillating incubator (BS-2F, Shuibei Science Experimental Instrument Factory, Jintan District, Jiangsu, China). *E. coli* was coated in Luria–Bertani medium (LB) and cultured at 37 °C for 24 h. Subsequently, the bacteria were diluted with sterile normal saline to 10^7^ CFU/mL bacterial suspensions.

### 2.2. CSP Antibacterial Activity Assay

The AGAR diffusion method was modified [17] to determine the antibacterial activity of the CSPs based on the diameter of the antibacterial zone. The prepared bacterial suspension was uniformly coated with NA or LB media, as indicated in Section 2.1. A sterile filter paper with a diameter of 6 mm was soaked in 10–40 mg/mL CSP solution or sterile water (negative control) for 30 s. The filter paper was then placed on the surface of the medium, and culturing was carried out at 30 °C and 37 °C for 24 h. The minimum inhibitory concentration (MIC) and minimum bactericidal concentration (MBC) of the CSPs were determined using the double gradient dilution method. The bacterial suspension (200 μL) and CSPs (10 mg/mL) were added to the liquid medium (2 mL total volume), and the culture was shaken at 180 rpm for 24 h. Sterile water was used as a negative control, and sodium diacetate (SDA), potassium sorbate, and sodium nitrite were positive controls. The final concentrations of CSPs were 1× MIC and 2× MIC, using the same amount of bacterial suspension added to the liquid medium. The OD_600_ of the culture medium at 0, 2, 4, 6, 8, 10, 12, and 24 h was measured using a microplate reader (ELX-800, BIO-TEK, Winooski, VT, USA), and growth curves were plotted according to the absorbance of 600 nm [18].

### 2.3. Bacterial Cell Wall and Membrane Damage Assay

Alkaline phosphatase (AKP), lactate dehydrogenase (LDH) activity, and total protein (bicinchoninic acid [BCA]) were measured in the treated bacterial supernatants according to the respective kit instructions (AKFA018C, AKCO003C, AKPR017, Beijing Box Shengong Technology Co., Ltd., Beijing, China). More specifically, 1 mL of CSP solution containing the equivalent of 1× MIC or 2× MIC was added to 10^4^ CFU/mL of bacterial suspension for 8 h. The suspension was then centrifuged at 8000× *g* for 10 min at 4 °C (H2050-R, Xiangtan Xiangyi Instrument Co., Ltd., Xiangtan, China), and the supernatant was tested. Sterile water was used as a negative control, and SDA as a positive control.

### 2.4. Scanning Electron Microscopy (SEM)

The 2× MIC CSPs solution (or sterile water as the negative control and SDA as the positive control) and 10^7^ CFU/mL bacterial suspension were combined in equal volumes, and the mixture was cultured for 8 h and then centrifuged (8000× *g* for 15 min at 4 °C). The precipitate was fixed in 2.5% glutaraldehyde for 12 h, re-suspended twice with phosphate-buffered saline (PBS), and eluted using a 30, 70, 90, and 100% ethanol gradient, centrifuging for 10 min each time. The combined eluates were precipitated, anhydrous ethanol (0.5 mL) was added, and the samples were vacuum freeze-dried. Thallus morphology was observed using a scanning electron microscope (Inspect F50, FEI, Hillsboro, OR, USA) at a magnification of 25 K.

### 2.5. Transmission Electron Microscopy (TEM)

Sample pre-treatment was performed as described in Section 2.4. Morphological changes in the three food-spoilage bacteria after CSP treatment were observed using a transmission electron microscope (Tecnai G2 F20, FEI) (enlarged 25 K).

### 2.6. ATP Content and ATPase Activity Assay

Specific kits were used to measure ATP content (AKOP004M), Na^+^K^+^-ATPase (AKOP001M), and Ca^++^Mg^++^-ATPase (AKOP002M) activity, per the manufacturer’s instructions (Beijing Box Shenggong Technology Co., Ltd., Beijing, China).

### 2.7. Activity Measurements of Key Tricarboxylic Acid (TCA) Cycle Enzymes

Bacterial suspensions containing 5–10 million bacterial cells were mixed with different concentrations of CSPs for 8 h, and then centrifuged for 10 min to collect the precipitates. The activities of α-ketoglutarate dehydrogenase (α-KGDH) and isocitrate dehydrogenase (ICDHm) were determined using kits, according to the manufacturer’s instructions (AKAC010M and AKAC009M, respectively, Beijing Box Shenggong Technology Co., Ltd., Beijing, China).

### 2.8. RT-qPCR Assay

Total DNA was extracted from *B. subtilis*, *P. fragi*, and *E. coli* using a bacterial genome DNA rapid extraction kit (B518225, Shanghai Bioengineering Co., Ltd., Shanghai, China) and quantified with an ultraviolet spectrophotometer (NanoDrop 2000, Thermo Scientific, Waltham, MA, USA). The mRNA levels of the genes of interest (Table 1) were quantified using qPCR analysis (ExicyclerTM 96, BIONEER, Daejeon, Republic of Korea) under the following conditions: 95 °C for 5 min, 95 °C for 10 s, 60 °C for 15 s, and 72 °C for 15 s, followed by 40 cycles of 72 °C for 90 s, 40 °C for 1 min, melting point 60–94 °C, every 1 °C melting point for 1 s, and 25 °C for 1–2 min. The mRNA expression levels were quantified using the 2^−ΔΔCT^ method.

### 2.9. Molecular Docking

AutoDock Vina [19] was used for the molecular docking of isocitrate dehydrogenase (IDH1) and α-ketoglutarate dehydrogenase (OGDH) with compounds PB1 and PC1, respectively. The 2D structures were downloaded from PubChem (https://PubChem.ncbi.nlm.nih.gov, accessed on 23 December 2022), and the 3D structures of the proteins were downloaded from the Research Collaboratory for Structural Bioinformatics Protein Data Bank (RCSB PDB). The PDB IDs of IDH1 and OGDH were 4UMX and 7WGR, respectively. During docking, the protein structure was converted to a PDB partial charge (Q) and atom type (T) (PDBQT) file containing all polar residues with hydrogen. The compounds were also converted to PDBQT files with all keys rotatable. The Lamarque genetic algorithm was used to simulate flexible docking. The grid box for IDH1 was set to center_x = 11.335, center_y = 27.039, center_z = 80.837, size_x = 40, size_y = 40, and size_z = 40. The grid box for OGDH was set at center_x = 99.908, center_y = 121.999, center_z = 110.413, size_x = 40, size_y = 40, and size_z = 40. The model of the complex was analyzed using Discovery Studio [20]. Interactions between protein–ligand complexes were mapped using PyMol.

### 2.10. Statistical Analysis

SPSS software (version 20; IBM, Chicago, IL, USA) was used to analyse data. Duncan’s multiple comparison test was used for difference analysis. A *p*-value < 0.05 was considered statistically significant. Origin 2018 software (version 9.0; OriginLab, Bethesda, MD, USA) was used to plot the data. All experiments were repeated at least three times, and the data were presented as the mean ± standard deviation (SD), *n* = 3.

## 3. Results and Discussion

### 3.1. CSPs Inhibit the Activity of Three Food-Spoilage Bacteria

#### 3.1.1. Diameter Analysis of Bacteriostatic Zone

**Figure 2:**
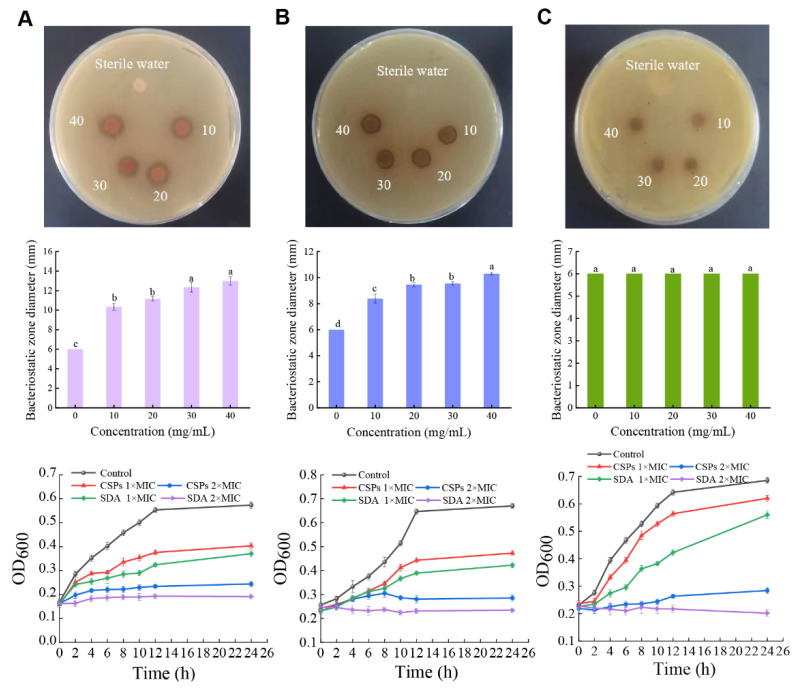
Bacteriostatic activity of chestnut shell polyphenols on food-spoilage bacteria and the growth curves. (**A**) *B. subtilis*; (**B**) *P. fragi*, and (**C**) *E. coli.* The diameter of the filter paper used in the experiment was 6 mm. Different lowercase letters within the same species indicate significant differences (*p* < 0.05) based on variance analysis. CSPs, chestnut shell polyphenols; sterile water, negative control. The 1× MIC and 2× MIC of CSPs for the three tested bacteria were 0.313 mg/mL and 0.625 mg/mL, respectively. The 1× MIC of SDA against *B. subtilis*, *P. fragi*, and *E. coli* was 0.625, 0.313, and 1.25 mg/mL, respectively, and the 2× MIC was 1.25, 0.625, and 2.5 mg/mL, respectively. CSPs, chestnut shell polyphenols; SDA, sodium diacetate; MIC, minimum inhibitory concentration.

The AGAR diffusion method can measure the bacteriostatic effects of applied samples. The antibacterial zone diameters of CSPs (40 mg/mL) against *B. subtilis*, *P. fragi*, and *E. coli* were 13.00 ± 0.42 mm, 10.30 ± 0.07 mm, and 6.00 mm, respectively (Figure 2). Sterile water did not produce a bacteriostatic effect on the three tested bacteria. CSPs had the best inhibitory effect on *B*. *subtilis*, followed by *P. fragi*, while *E*. *coli* did not produce a bacteriostatic zone. Similarly, Nunes et al. [21] found that *E*. *coli* treated with the polyphenol extract of Portuguese red wine at different concentration gradients did not generate an inhibitory zone. This may be related to bacterial structure. That is, *E. coli* and *P. fragi* were not inhibited or weakly inhibited because they are gram-negative with double-layer cell membranes, preventing most small molecules from crossing the outer membrane and aggregating in the cell, making the drug less active [22]. In contrast, *B*. *subtilis* is a gram-positive bacterium with a simple cell wall structure composed mainly of peptidoglycan and teichoic acid, resulting in greater inhibition than gram-negative bacteria [23]. This was further demonstrated by Snoussi et al. [24] who assessed the antibacterial activity of *D. flabellifolia* hydroalcoholic extract (3 mg/disc), revealing an antibacterial zone diameter of 8.00 ± 0.00 mm for the gram-negative bacterium *Enterobacter cloacae* and 36.33 ± 0.58 mm for the gram-positive bacterium *Staphylococcus epidermidis*. Meanwhile, a microshoot extract of *Nasturtium officinale* exhibits narrow-spectrum antibacterial activity against gram-positive bacteria and no inhibitory activity against gram-negative bacteria [25]. Similarly, although phenolic compounds extracted from geranium showed significant antibacterial activity against gram-positive and gram-negative bacteria, the effect was more considerable against gram-positive bacteria [26]. Therefore, CSPs may be used as natural preservatives to protect against food spoilage primarily caused by gram-positive bacteria, such as *B. subtilis*, including in fresh meat, dried bean curd in soy products, and thin Venetian veneers.

#### 3.1.2. MIC and MBC Analysis

The growth of *E. coli* under the filter paper of CSP treatment groups was inhibited, indicating that CSPs had an inhibitory effect on *E. coli.* The MIC of CSPs against the three tested bacteria was 0.313 mg/mL (Table 2). Under the same conditions, the MIC range of the positive control (SDA) was 0.313–5 mg/mL, depending on the type of bacterium. The MBC results showed that CSPs, with an MBC of 0.625 mg/mL, were more effective against the three tested bacteria than were the positive controls (SDA, potassium sorbate, and sodium nitrite), with an MBC range of 5–10 mg/mL. Among the positive controls, SDA showed the best inhibitory effect (Table 3). The inhibitory effects (MBC) of CSPs against *B. subtilis*, *P. fragi*, and *E. coli* were 16-, 16-, and 8-times that of SDA, respectively, suggesting that CSPs have natural preservative potential. Meanwhile, Zhao et al. [27] assessed the antibacterial effect of tea saponin derived from oil tea shell on *E*. *coli* and reported its MIC as 1 mg/mL and MBC as 4 mg/mL. Compared with the effect of CSPs on *E. coli*, the MIC and MBC of tea saponin were inferior. This may be related to the structure, composition, and purity of the polyphenols. SDA, potassium sorbate, and sodium nitrite are commonly used as food chemical preservatives, among which, sodium nitrite is not only anticorrosive but also impacts coloring and is thus more commonly used in meat products. Chestnut shells contain a large amount of brown pigment and is, thus, also used as a food colorant [28]. If applied to biscuits, chocolate cakes, and meat products, extracts of chestnut shells can serve as both a preservative and colorant, demonstrating their potential in food applications.

#### 3.1.3. Influence of CSPs on the Growth Curve of Food-Spoilage Bacteria

Microorganisms typically exhibit an “S”-shaped growth curve, generally divided into a hysteretic period, logarithmic growth period, stable period, and decline period [29]. The growth curves of *B. subtilis*, *P. fragi*, and *E. coli*, following treatment with saline (control), showed clear logarithmic and stable growth periods, consistent with normal bacterial growth (Figure 2A–C). However, the growth curves of the three bacteria were altered after CSP treatment. The growth curves of *P. fragi* and *E. coli* showed obvious retardation, whereas that of *B. subtilis* did not. Compared with the control group, the logarithmic growth stages of the three bacteria lagged following treatment with 1× MIC CSP. At 2× MIC CSP, the growth curve of the three bacteria was slowed, and the growth inhibition effect was more obvious. Similarly, Li et al. [30] combined chitosan with gallic acid (CTS-GA) and assessed its bactericidal effect on *E*. *coli* by assessing changes in its growth curve. The growth of *E. coli* was significantly inhibited when cultured under 1× MIC (*p* < 0.01). Moreover, in the current study, the inhibitory effect of 2× MIC CSP was significantly higher than that of 1× MIC SDA for the three bacteria. At 2× MIC CSP, relatively no reproductive growth was observed. Meanwhile, CSPs significantly inhibited *E. coli* compared with the control group (Figure 2C). This result contradicted the *E. coli* bacteriostatic zone findings and may be due to the culture time and CSP treatment method. That is, the CSP content absorbed by the filter paper of the bacteriostatic zone was uncertain. The growth curve adopted quantitative CSPs mixed with bacterial suspension for 8 h. Therefore, compared with the results of the bacteriostatic zone, the growth curve had a more obvious inhibitory effect on *E. coli.* These results showed that CSPs could inhibit the growth and propagation of the three tested bacteria; higher CSP concentrations corresponded with more obvious inhibitory effects.

### 3.2. CSPs Damage the Structure of the Bacterial Cell Wall and Membrane

**Figure 3:**
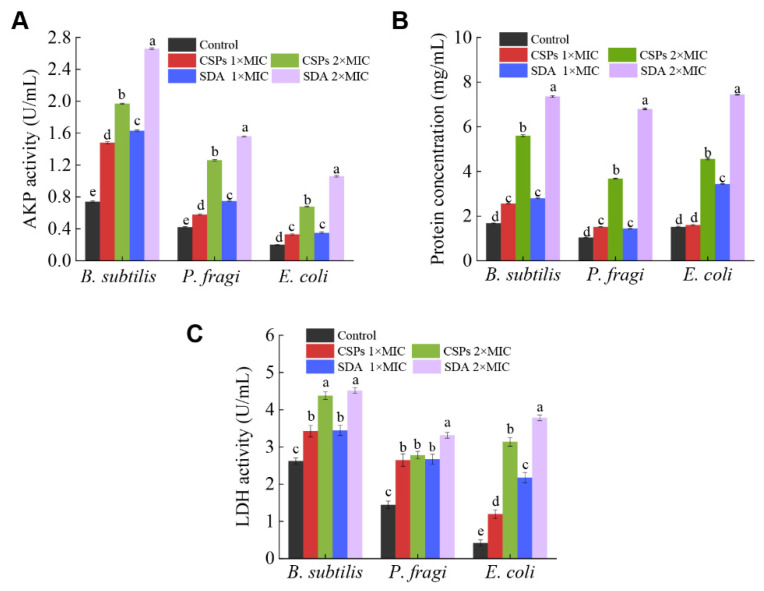
Enzyme activity and protein concentration after chestnut shell polyphenol (CSPs) treatment of three different bacteria. The three bacteria were treated with 1× MIC and 2× MIC of CSPs or sodium diacetate (SDA) for 8 h and the supernatants assayed. (**A**) Alkaline phosphatase (AKP) activity, (**B**) protein leakage, and (**C**) lactate dehydrogenase (LDH) activity. The CSP 1× MIC and 2× MIC for the three tested bacteria were 0.313 mg/mL and 0.625 mg/mL, respectively. The SDA 1× MIC against *B. subtilis*, *P. fragi*, and *E. coli* was 0.625, 0.313, and 1.25 mg/mL, respectively, and the 2× MIC was 1.25, 0.625, and 2.5 mg/mL, respectively. Different lowercase letters within the same species indicate a significant difference (*p* < 0.05).

AKP is a protease located between the bacterial cell membrane and cell wall. When the cell wall is damaged, a relatively large amount of AKP escapes, resulting in apoptosis. Therefore, the degree of cell wall integrity can be estimated by measuring the activity of AKP in the supernatant before and after treatment [31,32]. Figure 3A shows that, compared with the control group, AKP activity in the CSP-treated experimental group significantly increased in a dose-dependent manner (*p* < 0.05). The activity of AKP in the supernatant of *B. subtilis* treated with 1× and 2× MIC CSP was 2- and 2.66-times higher than that of the control group, respectively. The AKP activity of *P. fragi* increased from 0.58 to 1.26 U/mL after treatment with different CSP concentrations, while the AKP activity of *E. coli* treated with CSPs or SDA exhibited no significant change at 1× MIC (*p* > 0.05). The results showed that treatment with different concentrations of CSPs damaged the three tested bacteria to different degrees; in particular, the 2× MIC damaged the cell wall of all three bacteria, causing content leakage. Due to the increased concentration of AKP in the supernatants, the bacteriostatic effect of CSPs is likely related to cell wall destruction, which is consistent with the findings of Lin et al. [33].

LDH activity and protein leakage of the three bacteria were significantly increased after CSP treatment (*p* < 0.05; Figure 3B,C). At 1× MIC of CSPs, the protein released by *B. subtilis* significantly increased by 34% compared with the control group (*p* < 0.05). However, there was no significant change in the protein released by *E. coli* compared with the control group (*p* > 0.05), which may be related to differences in bacterial structure, confirming the conclusion made in Section 3.1.1. At 2× MIC CSP, the leaked protein content of *B. subtilis*, *P. fragi*, and *E. coli* was 3.3-, 3.6-, and 3.3-times that of the control group, respectively. The LDH activity of all three bacteria treated with different concentrations of CSPs was significantly higher than that of the control group (*p* < 0.05; Figure 3C). At 1× MIC, there was no significant difference in the LDH activity between *B. subtilis* and *P. fragi* treated with CSPs or SDA (*p* > 0.05). The LDH activity of *E. coli* treated with 1× MIC of SDA was 45% higher than that of *E. coli* treated with CSPs. At 2× MIC, the LDH activity of *B. subtilis* treated with CSPs or SDA did not differ significantly (*p* > 0.05), indicating that CSPs at 2× MIC had the best bacteriostatic effect on *B. subtilis*. This result is consistent with the results of the BCA protein leakage assay.

The cell membrane is another important structure that determines the integrity of bacterial cells [34]. Hence, we assessed the extent of cell membrane damage based on LDH activity and protein leakage [35]. Polyphenols interact with proteins, altering the structure of the plasma membrane and leading to the rapid loss of proteins, which can affect energy metabolism [36,37]. Our results indicated that CSPs caused irreparable damage to the bacterial cell membrane, resulting in the release of AKP, protein, and LDH, while interfering with the normal growth of the tested microorganisms. Zhao et al. [38] reported that treatment of *Staphylococcus aureus* with a 3× MIC of sugarcane (*Saccharum officinarum* L.) bagasse extract reduced the bacterial protein content and the inhibited growth and metabolism of the bacterial cells, findings which were consistent with our results.

### 3.3. CSPs Damage Bacterial Morphology

**Figure 4:**
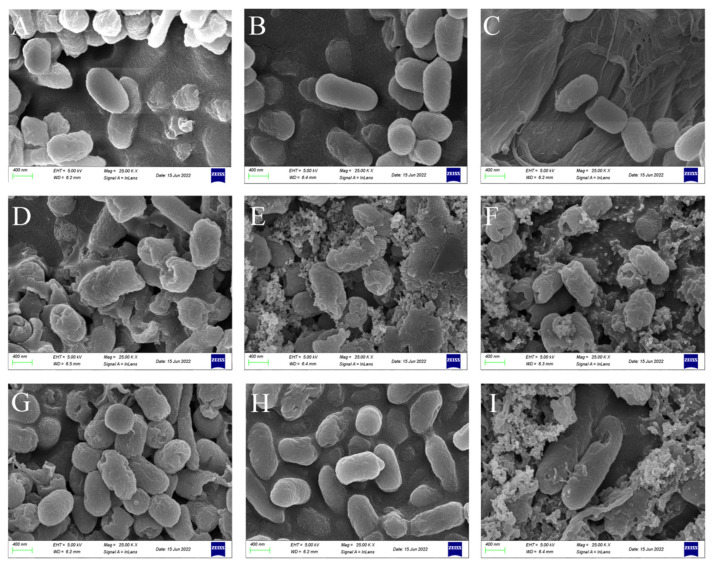
SEM (25,000×) images of the morphology of the three tested bacteria. (**A**–**C**) Controls; (**D**–**F**) 2× MIC CSP-treated group; and (**G**–**I**) 2× MIC SDA-treated group. (**A**,**D**,**G**) *B. subtilis*; (**B**,**E**,**H**) *P. fragi*; and (**C**,**F**,**I**) *E. coli*. Scale bar: 400 nm. The CSP 2× MIC against the three tested bacteria was 0.625 mg/mL. The SDA 2× MIC against *B. subtilis*, *P. fragi*, and *E. coli* was 1.25, 0.625, and 2.5 mg/mL, respectively.

To further investigate the mechanism of bacterial inhibition by CSPs, SEM and TEM were used to observe changes in cell morphology and ultrastructure. SEM images of control *B. subtilis*, *P. fragi*, and *E. coli* revealed regular rod-shaped morphologies, with smooth surfaces and clear and complete cell walls (Figure 4A–C). Compared with the control groups, the cell surface of the three tested bacteria treated with 2× MIC CSP showed varying degrees of damage (Figure 4D–F), with obvious cracks, dents, and adhesions. Similarly, at 2× MIC SDA, the cracks, adhesions, fractures, and fragmentation of cells were observed (Figure 4G–I).

**Figure 5:**
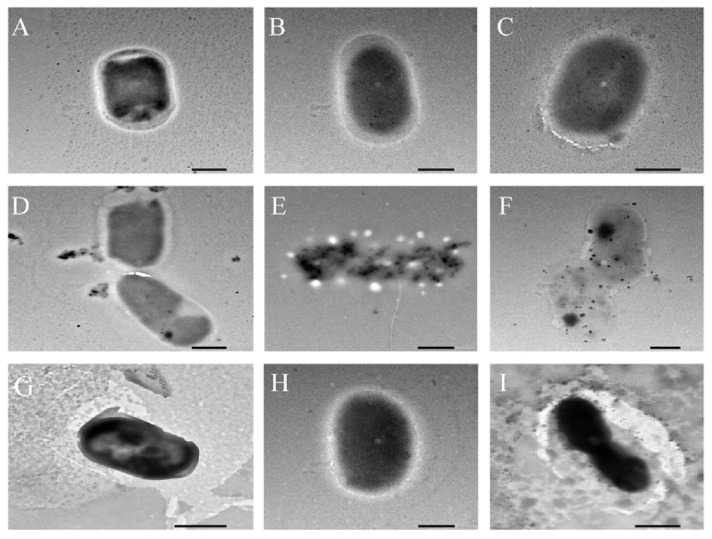
TEM (25,000×) images of the morphology of the three tested bacteria. (**A**–**C**) Controls; (**D**–**F**) 2× MIC CSP-treated group; and (**G**–**I**) 2× MIC SDA-treated group. (**A**,**D**,**G**) *B. subtilis*; (**B**,**E**,**H**) *P. fragi*; and (**C**,**F**,**I**) *E. coli*. Scale bar: 500 nm. The CSP 2× MIC against the three bacteria was 0.625 mg/mL. The SDA 2×MIC against *B. subtilis*, *P. fragi*, and *E. coli* was 1.25, 0.625, and 2.5 mg/mL, respectively.

To investigate the effect of CSPs on the internal structure of the bacteria, TEM observations were carried out (Figure 5), which verified the SEM conclusions. In the control group, there was no damage or voids on the cell surface of the three tested bacteria; the cell wall and cell membrane were intact, and the cytoplasm was compact and uniform without any leakage of cell contents (Figure 5A–C). After treatment with 2× MIC CSP (Figure 5D–F) and 2× MIC SDA (Figure 5G–I), the three types of bacterial cells appeared severely damaged: the cell wall and membrane were damaged, certain cell membrane structures were discontinuous, cell content leakage was severe, there were more particles on the cytoplasmic edge, and the cell contents appeared condensed. Compared with the control bacterial groups, the 2× MIC CSP treatment groups showed an obvious cavity formation phenomenon and many black clumps in the cells. Similarly, Alshuniaber et al. [39] found that *Staphylococcus aureus* and *E. coli* treated with extracted *Spirulina* polyphenols exhibited deformation, cell damage, and cell wall dissolution. By affecting the composition of the cell membrane, polyphenolic compounds alter the composition of fatty acids in bacterial cell membranes, resulting in significant changes in long-chain unsaturated fatty acids, thereby reducing membrane viscosity, inhibiting the synthesis of ergozite, reducing the fluidity of cell membranes, and increasing cell membrane permeability [40], which is consistent with the experimental results of this study.

Therefore, we concluded that the inhibition mechanism of CSPs on the three bacteria included damage to the cell wall and membrane structures, resulting in the aggregation and leakage of AKP, LDH, and other proteins. This damage affected the growth and metabolism of the bacteria and ultimately led to their death due to being unable to maintain their original cell morphology.

### 3.4. CSPs Reduce the Content of ATP and the Activity of ATPases

**Figure 6:**
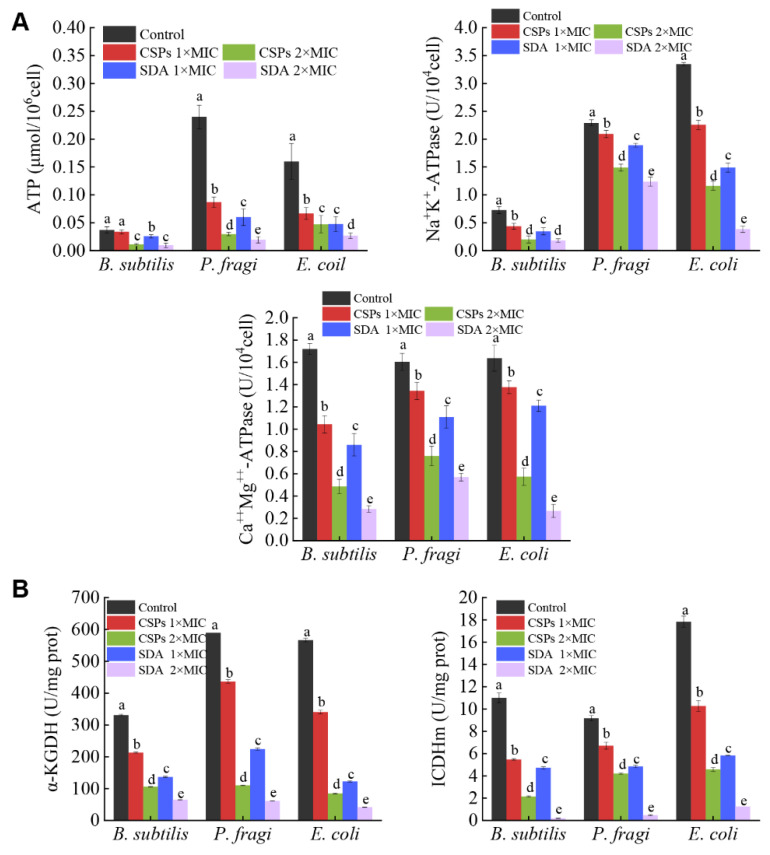
Effect of chestnut shell polyphenols (CSPs) on the breathing and energy metabolism in the three tested bacteria. (**A**) CSPs on the content of ATP and activity of ATPases. (**B**) CSPs on the activity of key enzymes in the TCA cycle. The CSP 1× MIC and 2× MIC against the three bacteria were 0.313 mg/mL and 0.625 mg/mL, respectively. The SDA 1× MIC against *B. subtilis*, *P. fragi*, and *E. coli* was 0.625, 0.313, and 1.25 mg/mL, respectively, and the 2× MIC was 1.25, 0.625, and 2.5 mg/mL, respectively. Different lowercase letters within the same species indicate significant differences (*p* < 0.05).

ATP is largely the product of oxidative phosphorylation, which can directly provide energy to living organisms. ATP content is typically stable, however, it decreases rapidly when the cell membrane is damaged or cells die; therefore, the ATP content can be used to reflect the number of living cells [41]. The ATP content of the bacteria was negatively correlated with the CSP concentration (Figure 6A). In *B. subtilis*, the intracellular ATP content decreased by 8.1% and 70.3% after treatment with 1× and 2× MIC CSP, respectively, compared with the control group. In turn, ATP content decreased by 29.7% and 75.7% after treatment with 1× and 2× MIC of SDA, respectively, compared with the control group. The ATP content in *P. fragi* and *E. coli* was similar to that in *B. subtilis*. Compared with the control groups, the ATP content in the CSP treatment groups decreased significantly (*p* < 0.05), and the effect on the three bacteria of the 2× MIC high-dose vs. the 1× MIC low-dose was significantly stronger (*p* < 0.05), showing a concentration-dependent trend. These results showed that CSPs inhibited ATP synthesis, and the bacteria likely died due to insufficient energy supplies. Similarly, the bacteriostatic mechanism of eugenic acid against *Cronobacter sakazakii* is related to a decrease in intracellular ATP content [42].

Na^+^K^+^-ATPase and Ca^++^Mg^++^-ATPase are proteases in biofilms that maintain the balance of ions inside and outside the cells and catalyze the synthesis and hydrolysis of ATP for energy. Both cell membrane damage and ion leakage affect the energy conversion system associated with the cell membrane [43]. The effects of different concentrations of CSPs on the ATPase activity of *B. subtilis*, *P. fragi*, and *E. coli* showed a trend consistent with that of the ATP content. With increased CSP concentration, the activities of Na^+^K^+^-ATPase and Ca^++^Mg^++^-ATPase decreased in the bacteria. Interestingly, in *B. subtilis*, the Na^+^K^+^-ATPase activity (0.20 U/10^4^ cell) following treatment with 2× MIC CSP was not significantly different (*p* > 0.05) from that measured after treatment with 2× MIC SDA.

The Ca^++^Mg^++^-ATPase activity of the three bacteria was significantly decreased after treatment with different concentrations of CSPs and SDA, compared with that of the untreated group (*p* < 0.05). Treatment with 2× MIC CSPs significantly decreased enzyme activity by 44.2%, 31.5%, and 52.9%, respectively, compared with that following treatment with 1× MIC of SDA (*p* < 0.05). These results indicate that CSPs inhibited the activities of Na^+^K^+^-ATPase and Ca^++^Mg^++^-ATPase by changing the permeability of the cell membrane, resulting in the failure of the ATP energy supply and the death of the bacterial cells, which may be another mechanism of CSP inhibition. Polyphenolic compounds, such as mulberry pigment, silymarin, baicalin, and silybin, can completely inhibit *E. coli* ATP synthase, whereas hesperidin, kaempferol, apigenin, and rutin have relatively weak inhibitory abilities (approximately 40–60%) [44], indicating that the structure of the phenol hydroxyl group is a key factor in bacteriostasis.

### 3.5. CSPs Inhibit the Activity of Key TCA Cycle Enzymes in Bacteria

The TCA cycle provides biological energy for cell survival by oxidizing nutrients [45]. Two key enzymes of the TCA cycle are α-KGDH and ICDHm. α-KGDH catalyzes the oxidation and decarboxylation of α-ketoglutaric acid to produce succinyl CoA and NADH in the TCA cycle; thus, the enzyme plays an important role in carbon, amino acid, and energy metabolism. In the absence of α-KGDH activity, α-ketoglutarate accumulates, posing a lethal threat to obligate aerobic cells [46]. Meanwhile, ICDHm catalyzes the oxidative decarboxylation of isocitrate to α-ketoglutaric acid and reduces NAD to NADH in the TCA cycle; thus, the enzyme plays an important role in metabolism, synthesis, and antioxidant stress. α-KGDH and ICDHm activity was inhibited in a concentration-dependent manner in the three CSP-treated bacteria (Figure 6B). Compared with the control group, α-KGDH activity decreased significantly in the three bacteria (B. subtilis; P. fragi, and E. coli.) treated with CSPs or SDA (*p* < 0.05). Treatment with CSPs at 2× MIC decreased enzyme activity by 22.5%, 50.8%, and 31% compared with SDA treatment at 1× MIC (*p* < 0.05). The activity of ICDHm was similar to that of α-KGDH. In *P. fragi*, there was no significant difference in the ICDHm activity in samples treated with 2× MIC CSPs or 1× MIC SDA (*p* > 0.05). Oregano essential oil can inhibit the respiratory metabolism of *S. aureus* by affecting the synthesis of metabolic products and the activity of key enzymes of the TCA cycle [47], suggesting that this may be another mechanism of bacterial inhibition by CSPs.

### 3.6. CSPs Regulate the Expression of Key TCA Cycle-Related Genes

In the TCA pathway, detrimental changes in the expression of genes encoding key enzymes may lead to an increase in reactive oxygen species, at which time the signaling pathway associated with apoptosis is activated, ultimately leading to bacterial cell apoptosis [48]. To further investigate the inhibitory mechanism of CSPs on the three tested bacterial species, the expression levels of 12 TCA cycle-related genes were determined using RT-qPCR (Table 3). In *B. subtilis*, the expression of *citA* (citrate synthase), *icd* (ICDHm), *odhA* (α-KGDH), and *atpA* (ATP synthase) was significantly downregulated in the CSP-treated groups compared with the control (*p* < 0.05). The expression of *icd* was downregulated by 40% after treatment with 1× MIC CSP and 70% (maximum extent) at 2× MIC, compared with the control group (*p* < 0.05).

In *P. fragi*, the expression of *gltA* (citrate synthase), *IS178_RS16090* (ICDHm), *IS178_RS16290* (α-KGDH), and *atpA* (ATP synthase) was significantly downregulated in the CSP-treated groups compared with the control group (*p* < 0.05). The expression of *IS178_RS16290* was downregulated by 49% after treatment with 1× MIC CSP, and the expression of *gltA* was downregulated by 71% after treatment with 2× MIC CSP, compared with the control.

In *E. coli*, the expression of *gltA* (citrate synthase), *icd* (ICDHm), *sucA* (α-KGDH), and *atpA* (ATP synthase) was significantly decreased in the CSP-treated groups by 17–65% compared with the control (*p* < 0.05). The downregulation of the four genes after treatment with 2× MIC CSP was superior to that following treatment with 1× MIC SDA, and the antibacterial effect was stronger. Among the four genes, CSPs showed the strongest inhibitory effect on *sucA*, which encodes α-KGDH.

The gene expression results indicate that CSP treatment mainly affected α-KGDH and ICDHm in the TCA cycle, resulting in abnormal gene expression and the inhibition of respiratory metabolism. This suggests that CSPs can destroy the bacterial cell structure at the molecular level by affecting gene expression and key enzyme activity, resulting in energy metabolism obstruction and cell death.

### 3.7. Molecular Docking Simulation of Major CSPs Components and Key TCA Cycle Enzymes

Simulated models of molecular docking for PB1 and PC1 with IDH1 and OGDH were used to study the main components and potential mechanism of the inhibition of CSPs on cell membrane dehydrogenase activity. The docking scores of PB1 with isocitrate dehydrogenase and α-ketoglutarate dehydrogenase were −11.7211 kcal/mol and −11.2414 kcal/mol, respectively. The docking scores of PC1 with isocitrate dehydrogenase and α-ketoglutarate dehydrogenase were −14.0050 kcal/mol and −12.8227 kcal/mol, respectively. The more negative the docking score, the better the binding affinity between the ligand and receptor [20]. The results showed that PB1 and PC1 could bind tightly to the bacterial cell membrane enzymes IDH1 and OGDH.

**Figure 7:**
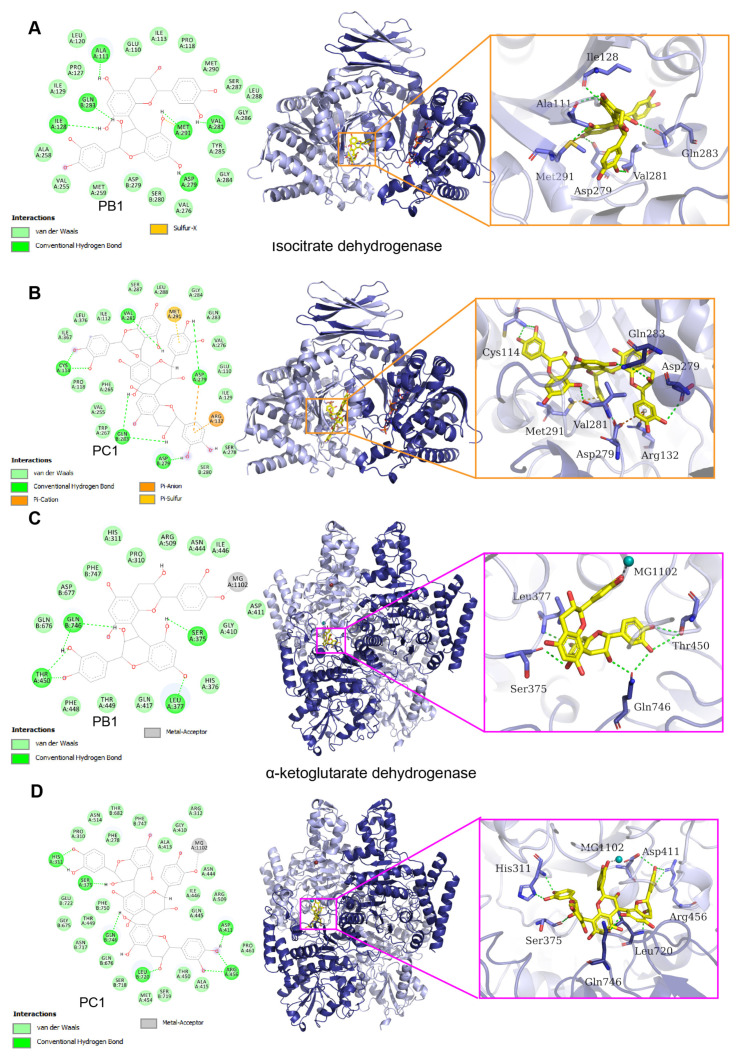
Two-dimensional cartoon and three-dimensional binding model of dehydrogenases and procyanidin B1 and C1. (**A**) Isocitrate dehydrogenase (IDH1) and procyanidin B1; (**B**) IDH1 and procyanidin C1; (**C**) α-ketoglutarate dehydrogenase (OGDH) and procyanidin B1; (**D**) OGDH and procyanidin C1. Yellow: procyanidin B1 and C1; light blue: IDH1 and OGDH chain A; dark blue: IDH1 and OGDH chain B; light blue sticks: residues in IDH1 chains A and B; dark blue sticks: residues in OGDH chains A and B; green dash line: hydrogen bond; orange dashed line: electrostatic interaction; pale yellow dashed line: sulfur-X interactions; gray dashed line: metal acceptors.

PB1 and PC1 are spatially complementary to the binding sites of IDH1 and OGDH (Figure 7). IDH1 and PB1 form hydrogen bonds, as well as sulfur-X, hydrophobic, and van der Waals (VDW) interactions (Figure 7A). The interactions between PB1 and IDH1 chain A residues Ile128, Ala111, Met291, Asp279, and Val281 are formed by hydrogen bonding; PB1 interacts with IDH1 chain B residue Gln83 through a sulfur-X interaction and with IDH1 chain A residues Trp124, Ala111, Ile128, Ile130, Ala282, Met291, and Val281 through hydrophobic interactions.

IDH1 and PC1 form hydrogen bonds, as well as electrostatic (pi-cation and pi-anion), Pi-sulfur, hydrophobic, and VDW interactions (Figure 7B). Hydrogen bond interactions form between PC1 and IDH1 chain A residues Cys114, Val281, and Asp279; electrostatic interactions form between PC1 and IDH1 chain B residues Gln283 and Asp279; electrostatic interactions form between PC1 and IDH1 chain A residues Arg132 and Asp279; Pi-sulfur interactions form between PC1 and IDH1 chain A residue Met291; hydrophobic interactions form between PC1 and IDH1 chain A residues Trp124, Tyr285, Ala111, Leu120, Val281, Ile128, Ala258, Met291, Arg109, Ile130, Ala282, Ile113, and Ile117; VDW interactions form among PB1 and PC1 with surrounding residues in IDH1. These interactions contributed to the binding energies between IDH1, PB1, and PC1.

OGDH forms hydrogen bonds, as well as metal–acceptor, hydrophobic, and VDW interactions with PB1 and PC1 (Figure 7C,D). PB1 forms hydrogen bonds with OGDH chain A residues Thr450, Ser375, and Leu377, and the residue Gln746 in OGDH chain B. PB1 and PC1 form metal receptor interactions with OGDH chain A residue Mg^2+^ ion. PB1 forms hydrophobic interactions with OGDH chain A residues Phe278, His513, Leu377, and Ala412, and the residues Phe750 and Leu720 in OGDH chain B. PB1 and PC1 form VDW interactions with surrounding residues in OGDH. PC1 forms hydrogen bond interactions with OGDH chain A residues Arg456, Ser375, His311, and Asp411, and the residues Leu720 and Gln746 in OGDH chain B. PC1 forms hydrophobic interactions with OGDH chain A residues His790, Gly447, Asp451, Leu377, Ala412, Pro452, and Ala455 and OGDH chain B residue Leu720. The binding energies of OGDH to PB1 and PC1 were determined primarily by these interactions.

PB1, PC1, and other proanthocyanidins are polyphenolic flavonoids that inhibit the growth and metabolism of microorganisms. Proanthocyanidins can reduce the stability of bacterial cell membranes by inhibiting β-lactamase activity, thereby inhibiting microbial growth [49]. PB1 and PC1, the main components of CSPs, played important roles in destroying bacterial cell membranes and inhibiting bacterial growth in this study.

## 4. Conclusions

The extracted CSPs exhibit antibacterial activity against *B. subtilis*, *P. fragi*, and *E. coli*, among which, *B. subtilis* is the most susceptible, and the inhibitory effect on gram-positive bacteria is stronger than that on gram-negative bacteria. Our results suggest the following bacteriostatic mechanism of CSPs: the bacterial cell wall and plasma membrane are damaged, cellular structural integrity is diminished, the permeability barrier of the membrane is reduced, and the leakage of cell contents increases. These processes lead to the inhibition of TCA cycle-related gene expression, resulting in the reduced activity of key enzymes, hindered respiration and energy metabolism, and the inhibition of ATP synthesis, thus, inhibiting cell proliferation and leading to bacterial cell death. PB1 and PC1 are the main components of the CSPs that exert antibacterial effects. At the same dose, the bacteriostatic effect of the CSPs was superior to the positive control SDA. Hence, collectively, this study demonstrates the potential of CSP extract as a low-price natural food preservative that can alleviate the harmful effects of synthetic chemical preservatives, reduce the bacterial contamination of food, and provide a theoretical basis for the high-value-added use of CSPs. However, while this study demonstrates that CSPs have an inhibitory effect on bacteria, it remains necessary to determine whether they exert an inhibitory effect on molds and yeasts. Moreover, it is necessary to evaluate the dose safety for adding CSP extracts to food products and to conduct toxicology analyses.

## Figures and Tables

**Table 1:** Gene primers.

	Gene	Forward Primer (5′–3′)	Reverse Primer (5′–3′)
Reference genes	*rpsj*	GAAACGGCAAAACGTTCTGG	GTGTTGGGTTCACAATGTCG
	*16S rRNA*	GTCACCGGCAGTCTCCTTAG	ATTGGTGCCTTCGGGAACAT
	*GAPDH*	ACTTACGAGCAGATCAAAGC	AGTTTCACGAAGTTGTCGTT
*Bacillus subtilis*	*citA*	CGGTTGTTTCAGCATTGGGG	TAACGTGACTCGTGCCGAAA
	*icd*	GCCTTTTGCATACTCGATGCC	TTTACGCCGGAGAAAAGGCT
	*odhA*	TCGCACCGCCTACTTGATAC	CAAAGCGGATACCCGGTACA
	*atpA*	GACAATCCGGCATTGATCGC	GCTTACCGTGAGCTGTCCTT
*Pseudomonas fragi*	*gltA*	AGAAGACCTGCGACGAAGTG	CTCGATGAAGTACGGGTCGG
	*IS178_RS16090*	GCAAAAGGTCGAAGAAGGCG	GTCACCGAGATGGTGTCCAG
	*IS178_RS16290*	TGGTCGATTACAACCTGGGC	GACTCGTTGCTGTCCGAAGA
	*atpA*	ATTGTGCGGATTCACGGTCT	GACTGCATCAGTCTCGGTGT
*Escherichia coli*	*gltA*	ACCCGTCTGTTCCATGCTTT	TTGCGCGGGTAAACAAATGG
	*icd*	CGATGATCGCGTCACCAAAC	CCGAAATATGCCGGTCAGGA
	*sucA*	GTACCGTACCGCCAACTTCA	TATCGGTTCTGTTCGTGCCC
	*atpA*	GTGTTATCCGCATTCACGGC	TCCAGGATACGGCCAGTACA

**Table 2:** Minimum inhibitory concentration and minimum bactericidal concentration of chestnut shell polyphenols against three tested bacteria.

Treatment Method	MIC (mg/mL)			MBC (mg/mL)		
	*B. subtilis*	*P. fragi*	*E. coli*	*B. subtilis*	*P. fragi*	*E. coli*
CSPs	0.313	0.313	0.313	0.625	0.625	0.625
SDA	0.625	0.313	1.250	10	10	5
Potassium sorbate	5	2.5	5	10	10	10
Sodium nitrite	2.5	0.625	5	10	10	10

MIC, minimum inhibitory concentration; MBC, minimum bactericidal concentration; CSPs, chestnut shell polyphenols; SDA, sodium diacetate.

**Table 3:** Chestnut shell polyphenols regulate the expression of key TCA cycle-related genes as determined using RT-qPCR.

Strain	Gene	Control	CSPs 1× MIC	CSPs 2× MIC	SDA 1× MIC	SDA 2× MIC
*Bacillus subtilis*	*odhA*	1.00 ± 0.08 ^aA^	0.55 ± 0.05 ^bB^	0.40 ± 0.03 ^cA^	0.45 ± 0.05 ^cB^	0.30 ± 0.03 ^dB^
	*icd*	1.00 ± 0.11 ^aA^	0.60 ± 0.04 ^bB^	0.30 ± 0.01 ^dB^	0.48 ± 0.01 ^cB^	0.23 ± 0.01 ^dC^
	*citA*	1.00 ± 0.11 ^aA^	0.70 ± 0.05 ^bA^	0.42 ± 0.05 ^cA^	0.58 ± 0.07 ^bA^	0.40 ± 0.05 ^cA^
	*atpA*	1.00 ± 0.11 ^aA^	0.61 ± 0.06 ^bB^	0.41 ± 0.03 ^cA^	0.53 ± 0.04 ^bAB^	0.18 ± 0.01 ^dC^
*Pseudomonas fragi*	*gltA*	1.00 ± 0.04 ^aA^	0.60 ± 0.04 ^bC^	0.29 ± 0.02 ^dC^	0.39 ± 0.03 ^cB^	0.19 ± 0.01 ^eD^
	*IS178_RS16090*	1.00 ± 0.05 ^aA^	0.75 ± 0.01 ^bA^	0.37 ± 0.04 ^dAB^	0.54 ± 0.02 ^cA^	0.27 ± 0.02 ^eB^
	*IS178_RS16290*	1.00 ± 0.11 ^aA^	0.51 ± 0.04 ^bD^	0.34 ± 0.04 ^cBC^	0.40 ± 0.03 ^cB^	0.23 ± 0.03 ^dC^
	*atpA*	1.00 ± 0.06 ^aA^	0.67 ± 0.04 ^bB^	0.43 ± 0.01 ^dA^	0.52 ± 0.05 ^cA^	0.32 ± 0.03 ^eA^
*Escherichia coli*	*gltA*	1.00 ± 0.06 ^aA^	0.72 ± 0.08 ^bAB^	0.40 ± 0.01 ^dB^	0.51 ± 0.05 ^cBC^	0.28 ± 0.02 ^eB^
	*icd*	1.00 ± 0.06 ^aA^	0.68 ± 0.03 ^bB^	0.41 ± 0.04 ^dB^	0.53 ± 0.08 ^cAB^	0.24 ± 0.02 ^eBC^
	*sucA*	1.00 ± 0.05 ^aA^	0.61 ± 0.02 ^bB^	0.35 ± 0.03 ^dB^	0.41 ± 0.01 ^cC^	0.23 ± 0.01 ^eC^
	*atpA*	1.00 ± 0.05 ^aA^	0.83 ± 0.09 ^bA^	0.51 ± 0.05 ^cA^	0.62 ± 0.05 ^cA^	0.34 ± 0.04 ^dA^

Lowercase letters in the table indicate significant differences in the same gene within the same species. Capital letters indicate significant differences in different genes within the same species (*p* < 0.05). CSPs, chestnut shell polyphenols; MIC, minimum inhibitory concentration; SDA, sodium diacetate. Gene expression levels were quantified using the 2^−ΔΔCT^ method.

## Data Availability

The data used to support the findings of this study can be made available by the corresponding author upon request.

## Supplementary Materials

The following supporting information can be downloaded at: https://www.mdpi.com/article/10.3390/foods12173312/s1, Table S1: Major components of chestnut Castanea mollissima shell polyphenol extract (CSP).
