# Supplementary material for: Chestnut Shell Polyphenols Inhibit the Growth of Three Food-Spoilage Bacteria by Regulating Key Enzymes of Metabolism
Source: Foods. 2023 Sep 2;12(17):3312. doi: 10.3390/foods12173312 (PMC10486611; doi:10.3390/foods12173312)
Supplement: Supplementary file 1 [file foods-12-03312-s001.zip › foods-2546445-supplementary.pdf]

Supplement Table S1

Table S1 Major components of chestnut Castanea mollissima shell polyphenol extract (CSP)

Component	Content (mg/g)
Vanillic acid	0.26±0.01
Myricetin	0.77±0.02
Androgenic acid	0.27±0.01
Quercetin	1.64±0.05
Trans-4-hydroxy-3-methoxycinnamic acid	0.08±0.00
Trans-p-coumaric acid	0.09±0.00
3,4-dihydroxybenzoicacid	5.54±0.15
Gallic acid	11.20±0.79
Ellagic acid	1.15±0.03
Procyanidin C1	126.00±2.72
Procyanidin B1	148.00±2.57
Procyanidin B2	18.70±0.83
(+)-Catechin	46.90±1.12
(-)-Epicatechin	2.07±0.09
Quercetin 3-glucoside	0.32±0.01
(-)-Epicatechin gallate	1.73±0.06
(-)-Epigallocatechin gallate	0.09±0.00
Total	364.81±3.58

Values are expressed as the mean ± standard deviation, n = 3.
